# Endothelial-derived extracellular matrix ameliorate the stemness deprivation during *ex vivo* expansion of mouse bone marrow-derived mesenchymal stem cells

**DOI:** 10.1371/journal.pone.0184111

**Published:** 2017-08-30

**Authors:** Ming-Kang Lee, Shau-Ping Lin, Wei-Chun HuangFu, Dee-Shiuh Yang, I-Hsuan Liu

**Affiliations:** 1 Department of Animal Science and Technology, National Taiwan University, Taipei, Taiwan; 2 Institute of Biotechnology, National Taiwan University, Taipei, Taiwan; 3 Research Center for Developmental Biology and Regenerative Medicine, National Taiwan University, Taipei, Taiwan; 4 Agricultural Biotechnology Research Center, Academia Sinica, Taipei, Taiwan; 5 The Ph.D. Program for Cancer Biology and Drug Discovery, College of Medical Science and Technology, Taipei Medical University, Taipei, Taiwan; 6 School of Veterinary Medicine, National Taiwan University, National Taiwan University, Taipei, Taiwan; University of Cincinnati College of Medicine, UNITED STATES

## Abstract

Mesenchymal stem cells (MSCs) hold great potential in cell therapies by virtue of the regenerative effects and immunomodulatory properties, but the scarce nature of MSCs makes *ex vivo* expansion indispensable prior to transplantation purposes. However, potential loss of stemness ensuing culture expansion has hindered the advancements in MSCs-based treatments. In principle, stemness could be preserved by reconstructing the stem cell niche. To test whether the endothelial cells (ECs) participate in the constitution of the stem cell niche for mesenchymal stem cells (MSCs), ECs derivatives including extracellular matrix (ECM) and conditioned medium (CM) prepared from aortic endothelial cells (AECs) and Mile Sven 1 endothelial cell line (MS1) were investigated for the potential to maintain MSCs stemness. MSCs expanded on endothelial ECMs, especially on MS1-ECM, possessed a more juvenile morphology and showed delayed proliferation, when compared with untreated MSCs and MSCs on MSC-ECM and in CMs. Once induced, MS1-ECM group showed better tri-lineage differentiations indicating that MS1-ECM could better preserve MSC stemness. MSCs on MS1-ECM showed stronger immune-modulatory potential and had significantly higher H3K27me3 with lower *Kdm6b* expression. Taken together, MS1-ECM shapes an inhibitory chromatin signature and retains MSCs stemness. Our work provided supportive evidence that MSCs can reside in a perivascular niche, and a feasible novel approach for MSCs expansion.

## Introduction

The mesenchymal stem cells (MSCs) were first found in the bone marrow (BM), and are a small population of cells capable of self-renewal and possessing multi-lineage potential to differentiate into osteoblasts, chondrocytes and adipocytes [1–3]. In addition to the bone marrow, multiple origins of MSCs were also revealed, such as adipose tissue, skeletal muscle, amniotic fluid, etc. [4–6]. MSCs are highly heterogeneous and accordingly minimal criteria for defining human MSCs were recommended by International Society for Cellular Therapy as: first, MSCs must be plastic adherent when maintained in standard culture condition; second, MSCs must be positive for CD73, CD90 and CD105, and be negative for CD34, CD45, CD11b or CD14, CD79α or CD19 and class II major histocompatibility complex (MHCII); third, with proper induction, MSCs must be able to differentiate into osteoblasts, chondrocytes and adipocytes *in vitro* [7].

MSCs attract attentions in recent decade because they show promising beneficial effects in various health conditions and have been contemplated as an “injury drugstore” [8, 9]. MSCs are considered immune-privileged and hence ideal for cell therapies [10]. Furthermore, MSCs secret trophic factors and cytokines to promote cell proliferation and inhibit the occurrence of apoptosis. For example, MSCs transplantation improved proliferation of endogenous neural stem cells in subventricular zone and prevented apoptosis of new born cells which migrating to ischemic environment in a rat stroke model [11], while exosomes containing miR-10a secreted from amniotic fluid-derived MSCs ameliorated apoptosis of granulosa cells and ovarian follicular atresia after chemotherapy [12]. In addition, MSCs cast strong immune modulatory effects via immune cells such as dendritic cells, natural killer cells, and T-cells [13–17]. Accordingly, graft-versus-host disease (GvHD) is one of the most epitomic and encouraging MSCs-based clinical trials [18–20].

It is believed that MSCs only account for approximately 0.001% to 0.01% of whole nucleated cells isolated from bone marrow aspirates [3]. Previous studies showed that the mean nucleated cells of bone marrow aspirate from each patient ranged from 1.3×10^7^ to 9×10^7^ per mL [21, 22]. Accordingly, there were approximately only 130 to 9,000 of MSCs from each mL of freshly isolated bone marrow aspirates. In the clinical trials targeting GvHD, the MSCs infusion number ranged from 0.3×10^6^ to 10×10^6^ cells per kg of body weight, and more than one infusion was needed [23]. Consequently, the number of MSCs from freshly isolated marrow was far less sufficient for cell therapies. Thus, *ex vivo* expansion is indispensable and MSCs at passage up to 7 were seen in clinical trials [23]. Unfortunately, MSCs might gradually lose the core stem cells property during passaging.

The core stem cell property, also known as “stemness”, refers to the ability to self-renew and to generate differentiated progenies [24]. In MSCs, progressive loss of stemness could be observed by morphological change and decrease in proliferation and differentiation plasticity. Juvenile MSCs have spindle shape appearance with prominent long-short axis, but senescent cells flatten, enlarge in size and accumulate cell debris [25]. Late passages of MSCs severely lost the numbers of population doubling [26]. MSCs from aged donors also showed profound loss in colony-forming efficiency, and loss in population doublings accompanied with slower growth rate before reaching growth arrest [27, 28]. Senescent passage was reported with reduced adipogenic, osteogenic, and chondrogenic differentiation with feeble bone-forming efficiency compared to freshly isolated MSCs [25, 29]. In treating GvHD, patients infused with late passage (passage 3–4) MSCs developed lower complete response rate (38%) than patients infused with early passage (passage 1–2) MSCs (86%), while patients receiving early passage MSCs had significantly higher 1-year survival rate (75%) compared to late passage MSCs (21%) [30] suggesting that loss of stemness also compromise the immune modulatory effect of MSCs.

It was hypothesized that proper stemness maintenance relies on specific microenvironment [31], and it is now widely accepted that stem cells remain self-renewable and undifferentiated in their niche microenvironment. Emerging evidence suggested that MSCs might occupy a perivascular origin, while the pericytes might serve as one of the primitive origins of MSCs. Pericytes, also known as Rouget cells, mural cells and perivascular cells, share conventional MSCs characteristics [32, 33], while MSCs of various origins reside at perivascular locations [34, 35]. On the notion that perivascular microenvironment could be MSCs niche, several studies embarked on investigating the effects of endothelial cells (ECs) to MSCs. In a three-dimensional (3D) culture model, human MSCs were co-cultured with human umbilical vein endothelial cells (HUVECs) and the two populations of cells self-assembled into spheroids with organized partitioning. Furthermore, HUVECs directed quiescence of MSCs and enhanced osteogenic differentiation of MSCs after induction [36]. These discoveries supported the notion that ECs could possibly orchestrate the MSC niche. In this study, we further dissected the endothelial derivatives, including extracellular matrix (ECM) and soluble factors, and tested whether they were able to preserve MSCs stemness by evaluating the morphology, proliferation potential and tri-lineage differentiation of MSCs.

## Materials and methods

### Animals

ICR mice aged from 6–12 weeks were purchased from Laboratory Animal Center of National Taiwan University and euthanized to harvest cells for experiments. All experimental procedures on animals were reviewed and approved by the Institutional Animal Care and Use Committee (IACUC) of National Taiwan University (NTU-100-EL-1), and were performed in accordance with the approved guidelines.

### Isolation of mouse BM-MSCs

Mouse BM-MSCs were harvested as previously described [37, 38]. Briefly, mice were anesthetized with 2,2,2-Tribromoethanol (Avertin, 250 mg/Kg) and sacrificed by cervical dislocation. The femur and tibia were separated from mouse after euthanasia with muscle and connective tissue quickly removed by forceps and scissors. Culture medium Dulbecco's modified Eagle's medium (DMEM; Gibco, Grand Island, NY, USA) supplemented with 3.7 g of sodium bicarbonate (NaHCO_3_; Sigma-Aldrich, St. Louis, MO, USA), 20% of fetal bovine serum (FBS; Hyclone, Logan, UT, USA) and antibiotics (100 U/mL of penicillin and 100 μg/mL of streptomycin; Gibco) were used to flush out the bone marrow. Flushed-out bone marrow and minced femur and tibia were cultured on 100-mm petri dish (TPP, Trasadingen, CH) for 9 days with medium changed every 3 days. Cells were then enriched by a magnetic-activated cell sorting system (Miltenyi Biotec, Teterow, DE) using double negative selection with CD11b (Miltenyi Biotec) and CD45 (Miltenyi Biotec) microbeads according to manufacturer’s protocol to avoid the contamination of hematopoietic lineage. MSCs were then seeded at a density of 5×10^4^ cells per cm^2^. All MSCs among different niche-mimicking groups were cultured with Medium E (1:1 mixture of 20% FBS in DMEM and Medium G [DMEM containing 3.7 g of sodium bicarbonate, 20% of fetal bovine serum, 100 U/mL of penicillin, 100 μg/mL of streptomycin, 1× non-essential amino acid (Sigma-Aldrich), 25 mM HEPES buffer (Sigma-Aldrich), 100 μg/mL heparin and 100 μg/mL endothelial cells growth supplement (Millipore, Billerica, MA, USA)]) on the consideration that collecting conditioned-medium (CM) of aortic endothelial cells (AECs) required endothelial cell growth supplement. Cells were all seeded with a density of 5×10^4^ cells per cm^2^. Confluence could occur after 2 days and ready for passaging with same seeding density.

### Endothelial cell culture

The procedure to harvest mouse AECs was modified from a previous report [39]. Briefly, mouse thorax was opened after anesthesia to expose lungs and heart and was sacrificed by rapid exsanguination. The abdominal aorta was cut to release blood and 1 mL of phosphate-buffered saline (PBS; Amresco, Solon, USA) containing 1000 U/mL of heparin was injected into the left ventricle. The aorta was dissected from aorta arch to abdominal aorta and was immersed in 20% FBS-DMEM containing 1000 U/mL of heparin. After insertion of a 26-gauge needle into the distal side of aorta, the aortic lumen was washed with serum-free DMEM. The proximal aorta was ligated with silk thread, and the lumen was subsequently perfused with type II collagenase (2 mg/mL in serum-free DMEM; Sigma-Aldrich). After 45 mins of digestion at 4°C, cells were flushed out and centrifuged at 1,200 rpm for 5 mins. Cells were resuspended with DMEM containing 20% of FBS and were seeded on 35-mm petri dish (TPP) pre-coated with collagen I (Sigma-Aldrich). After 90 mins of incubation, the medium was replaced by Medium G to remove smooth muscle cells. AECs reached confluence after 9 days of culture with medium changed every 3 days.

MS1 pancreatic islet endothelial cell line (Bioresource Collection and Research Center, Hsinchu, TW) were cultured with high glucose DMEM (Gibco) supplemented with 5% FBS, 1.5 g of NaHCO_3,_ 100 U/mL of penicillin and 100 μg/mL of streptomycin with a 1:4 to 1:6 passaging ratio and were subcultured every 2 days.

## Preparation for collagen I-coated 6 well plate

Collagen I was dissolved in 0.1 M acetic acid (Merck, Darmstadt, DE) to obtain 0.1% (w/v) stock. The working concentration (0.01%) was obtained after 10-fold dilution. Coated plates with 6–10 μg/cm^2^ then allowed protein to bind under 37°C for 3 hrs. Excessive collagen I solution was discarded and the plates were exposed to UV light for sterilization. The plates were washed once with PBS immediately before introduced to cells.

### Preparations of cell-free extracellular matrix-coated plates

To test the effect of ECMs derived from AECs, MS1 cells and MSCs in preserving the stemness of MSCs, cell-free ECM-coated plate were prepared for the *ex vivo* expansion of MSCs [40]. AECs (passage 3 and 4) and MS1 cells (passage 9 through 13) were seeded on collagen I-coated 6 well plate. MS1 cells were seeded with 1:10 dilution, while seeding densities for MSCs (passage 3 and 4) and AECs were 2×10^4^ cells per cm^2^. Note that bone marrow cells without stringent enrichment or purification were used in the previous work to reconstitute a more native marrow ECM [40], while the MSCs we used here were not only at passage 3 and 4 but also enriched by negative selection during isolation so that the role of each type of cells in constituting the ECM could be clarified. Cells were cultured for 15 days with the medium changed twice a week. In the last 8 days of culture, additional 50 μM of ascorbic acid (Sigma-Aldrich) was supplemented to the culture medium. De-cellularization was carried out by treating the cells with 0.5% of Triton X-100 (Amresco) containing 20 mM of NH_4_OH (Riedel-de Haën, Seelze, DE) in PBS for 1 min at room temperature and then 25 μg/mL DNase I (Sigma-Aldrich) for 30 mins at room temperature. The ECM coated plates were rinsed with PBS twice and then could be stored in PBS containing 100 U/mL of penicillin, 100 μg/mL of streptomycin and 0.25 μg/mL of fungizone (Sigma-Aldrich) at 4°C for up to 3 weeks.

### Preparations of conditioned-medium

For the preparation of CM, 5×10^5^ AECs were seeded on 35-mm petri dish with Medium G for 1 day. AECs were then cultured in Medium E for additional 2 days as the AEC-CM. On the other hand, MS1 cells were seeded at 1:6 dilution in Medium E for 1 day as the MS1-CM. CMs were collected in tubes and preserved at -80°C.

### Quantification of long/short axis ratio

Since the spindle-shaped morphology is one of the signatures of MSCs, we used the long/short axis ratio as an index to assess the morphology of MSCs. For quantification of long/short axis ratio, 20 cells in each group with clear and unmasked contour were chosen as representatives. For each cell, the long and short axes were measured in ImageJ [41] to calculate the ratio. All extreme values (the greatest and the smallest value) in each group were depleted.

### MTT assay

For the observation of cell proliferation and viability, 1.5×10^3^ of MSCs in 100 μL culture medium were seeded into 96-well plate (TPP). Cells were incubated for 1, 5, 9, 12, 15 and 18 days respectively. A total of 10 μL of 3-(4, 5-Dimethylthiazol-2-yl)-2,5-diphenyltetrazolium bromide (MTT, Sigma) reagent (5 mg/mL) were added and incubated for 2 hrs at 37°C. MTT solvent was carefully removed and replaced by 100 μL of DMSO (D5879, Sigma-Aldrich). Absorbance at 570 nm was recorded with ELISA reader (SpectraMAX 190, Molecular Devices, Sunnyvale, CA, USA) and the cell numbers were calculated according to the optimal standard cell count curve.

### Osteogenic differentiation

Osteogenic induction medium was comprised of 10% FBS-DMEM with 0.1 μM dexamethasone (Sigma-Aldrich), 10 mM β-Glycerophosphate (Sigma-Aldrich) and 50 μM L-ascorbic acid 2-phosphate (Sigma-Aldrich) [42]. Each 5×10^4^ cells per cm^2^ were seeded into 6-well plates and confluent in 2 days. The cells were then introduced to osteogenic induction medium for 14 or 21 days with the medium changed every 3 days. To assess the calcium precipitation, cells were washed with PBS once and then fixed with 10% of formaldehyde (Sigma-Aldrich) for 10 mins with gentle shaking. After washed with PBS again, cells were stained with 500 μL of 2% Alizarin Red S (ARS, pH 4.1–4.3; Sigma-Aldrich) for 15 mins. After extensive PBS irrigation, precipitation was dissolved in 500 μL of 10% cetylpyridinium chloride (Sigma-Aldrich) in 8 mM Na_2_PO_4_ (Sigma-Aldrich) and 1.5 mM KH_2_PO_4_ (Sigma-Aldrich). The absorbance of 570 nm was recorded and quantity of calcium could be determined using optimal ARS standard curve.

### Adipogenic differentiation

The composition of adipogenic differentiation cocktail was 10% FBS-DMEM supplemented with 10 μg/mL insulin (Sigma-Aldrich), 1 μM dexamethasone, 0.5 μM isobutyl-methylxanthine (Sigma-Aldrich) and 100 μM indomethacin (Sigma-Aldrich) [3]. Similar to osteogenic induction, cells were introduced to 12-well plates (TPP) for 7 days of adipogenic differentiation along with medium renewal every 3 days. After fixed and washed, cells were immersed in 1 mL of propylene glycol (JT Baker, Center Valley, PA, USA) for 2 mins at room temperature and stained with 300 μL of 0.5% Oil Red O (Sigma-Aldrich) for 15 mins for neutral lipid. To quantify the results, the cells were immersed in 60% of propylene glycol (in water), washed with ultrapure water, and the Oil Red O was dissolved in 200 μL of DMSO. Quantification could be carried out by measuring the absorbance at 550 nm and standardized to optimal standard curve.

### Chondrogenic differentiation

The chondrogenic induction medium was composed of DMEM containing 1% of FBS, 10 ng/mL of transforming growth factor beta 1 (TGF-β1; R&D Systems, Minneapolis, MN, USA), 6.25 μg/mL of insulin and 50 nM of L-ascorbic acid 2-phosphate [43]. Each 2.5×10^5^ cells were collected in a tube for centrifugation at 1,200 rpm for 5 mins and incubated for 21 days in chondrogenic medium with medium change every 3 days. Cell aggregate could be observed after 3 days of culture. After 21 days of culture, the cell aggregates were fixed with 10% of formalin and prepared for paraffin section. The section slides were rehydrated by xylenes (JT Baker) wash and 100% ethanol to PBS gradient wash. To observe the accumulation of glycosaminoglycan (GAG) within the cell aggregates, slides were stained with 0.1% of Toluidine Blue O (Sigma-Aldrich) for 5 mins, washed with PBS, sealed and documented under microscope (DM 2500, Leica, Wetzlar, DE).

### Scanning electron microscope (SEM)

The procedure of SEM sample preparation was as described in a previous publication [44]. In brief, cell-free extracellular matrix-coated plates were prepared on 12-mm cover glasses. De-cellularized ECM were then washed with PBS twice and pre-fixed by 2.5% glutaraldehyde (Electron Microscopy Sciences, Hatfield, PA, USA) in 0.1M phosphate buffer (PB) for 30 mins at room temperature. After washed with 0.1M PB for 3 times 15 mins each, samples were post-fixed by 1% osmium tetroxide (OsO_4_; Electron Microscopy Sciences) in 0.1M PB for 30 mins. After rinsed with 0.1M PB, the samples were dehydrated by 30% to 100% ethanol gradient wash followed by 100% acetone (Macron Fine Chemicals, Center Valley, PA, USA) twice for 10 mins. For critical point drying (CPD), each sample was placed into a specimen cassette padded with filter paper, and the specimen cassette was filled with 100% acetone to top of the holder and transferred into the chamber (3–4°C) of a critical point dryer (Hitachi, Tokyo, JP). Liquid CO_2_ from siphon tank was administrated to completely replace the acetone prior to heating to the critical point under 80 atm (standard atmosphere). Samples on cover glasses were then adhered on the aluminum stubs with the aid of double-sided carbon tapes and placed on ion coater (IB-2, Eiko, Tokyo, JP). After achieving sufficient vacuum (index number below 2.5 when HV control at 5), voltage was introduced (HV at 7), and gold was coated on samples for 3 mins. Finally, the samples were observed with SEM (Inspect S, FEI, Hillsboro, Oregon, USA) at 20,000× magnifications.

### Quantitative PCR

To quantitatively analyze the gene expression levels, total RNA was harvested by using TRIzol Reagent (Invitrogen, Carlsbad, CA, USA) and GENEzol TriRNA Pure Kit (Geneaid, New Taipei City, TW) following the manufacturers’ instructions. DNA in the sample was removed with RNase-Free DNase I (Geneaid). Reverse transcription was performed with 150 or 300 ng RNA and SuperScript III reverse transcriptase (Invitrogen) according to the manufacturer’s instruction. Real-time PCR was performed with 10-fold diluted cDNA sample and iQ SYBR Green Supermix (BIO-RAD, Hercules, CA, USA) in a thermal cycler (CFX96 Real-Time System, BIO-RAD). Target genes were calibrated with internal control (*Gapdh*) and normalized to control group using ΔΔC_q_ method. The primer sequences used in this study were as listed (Table 1).

**Table 1:** Primers used in quantitative PCR.

Gene	Sequence (5’→ 3’)	Product size (bp)	Tm (°C)
*Gapdh*	Forward: CATGGCCTTCCGTGTTCCTA	55	60
	Reverse: GCGGCACGTCAGATCCA		
*iNos*	Forward: CAGCTGGGCTGTACAAACCTT	95	60
	Reverse: CATTGGAAGTGAAGCGGTTCG		
*Ezh2*	Forward: AAGCACAATGCAACACCAAA	169	60
	Reverse: AGACGGTGCCAGCAGTAAGT		
*Kdm6a*	Forward: ATCCCAGCTCAGCAGAAGTT	196	60
	Reverse: GGAGGAAAGAAAGCATCACG		
*Kdm6b*	Forward: CCCCCATTTCAGCTGACTAA	199	60
	Reverse: CTGGACCAAGGGGTGTGTT		

To mimic the inflammatory environment, cells were seeded with a density of 5×10^4^ cells per cm^2^. After 24 hrs of incubation, recombinant murine interferon-γ (PeproTech, Rehovot, IL) was introduced to cells with high (500 ng/mL) or low (100 ng/mL) concentrations and incubated for additional 24 hrs before the RNA was harvested.

### Western blot

To quantitatively evaluate the tri-methylation at lysine 27 of histone H3 (H3K27me3), cell lysates were harvested with 100 μL RIPA buffer (BIOMAN SCIENTIFIC, New Taipei City, TW). Total harvested proteins were quantified by BCA Protein Assay Kit (Thermo Scientific, Rockford, IL, USA) following manufacturer’s protocol. The protein samples were then separated by 12% SDS-polyacrylamide gel electrophoresis (PAGE), transferred onto a 0.22 μm PVDF membrane (Millipore), and probed with anti-lamin B antibody (Santa Cruz Biotechnology, Dallas, TX, USA) and anti-H3K27me3 antibody (Millipore) with anti-goat IgG HRP (1:5000; Abcam, Cambridge, UK) and goat anti-mouse IgG+IgM HRP (1:2000; Abcam) as secondary antibody. The signal was detected using chemiluminescence HRP substrate (Millipore) and documented by ChemiDoc Touch Imaging System (BIO-RAD) followed by manufacturer’s instruction.

### Statistical analysis

Statistical analysis was done in GraphPad Prism software. Data were assessed by one-way ANOVA with Tukey post-test and displayed as mean ± standard error of the mean (SEM). All samples for each experiment include 3 biological replicates except for MTT assay, which is 8 biological replicates. * signifies *p* < 0.05, ** *p* < 0.01, *** *p* < 0.001 and **** *p* < 0.0001.

## Results

**Fig 1:**
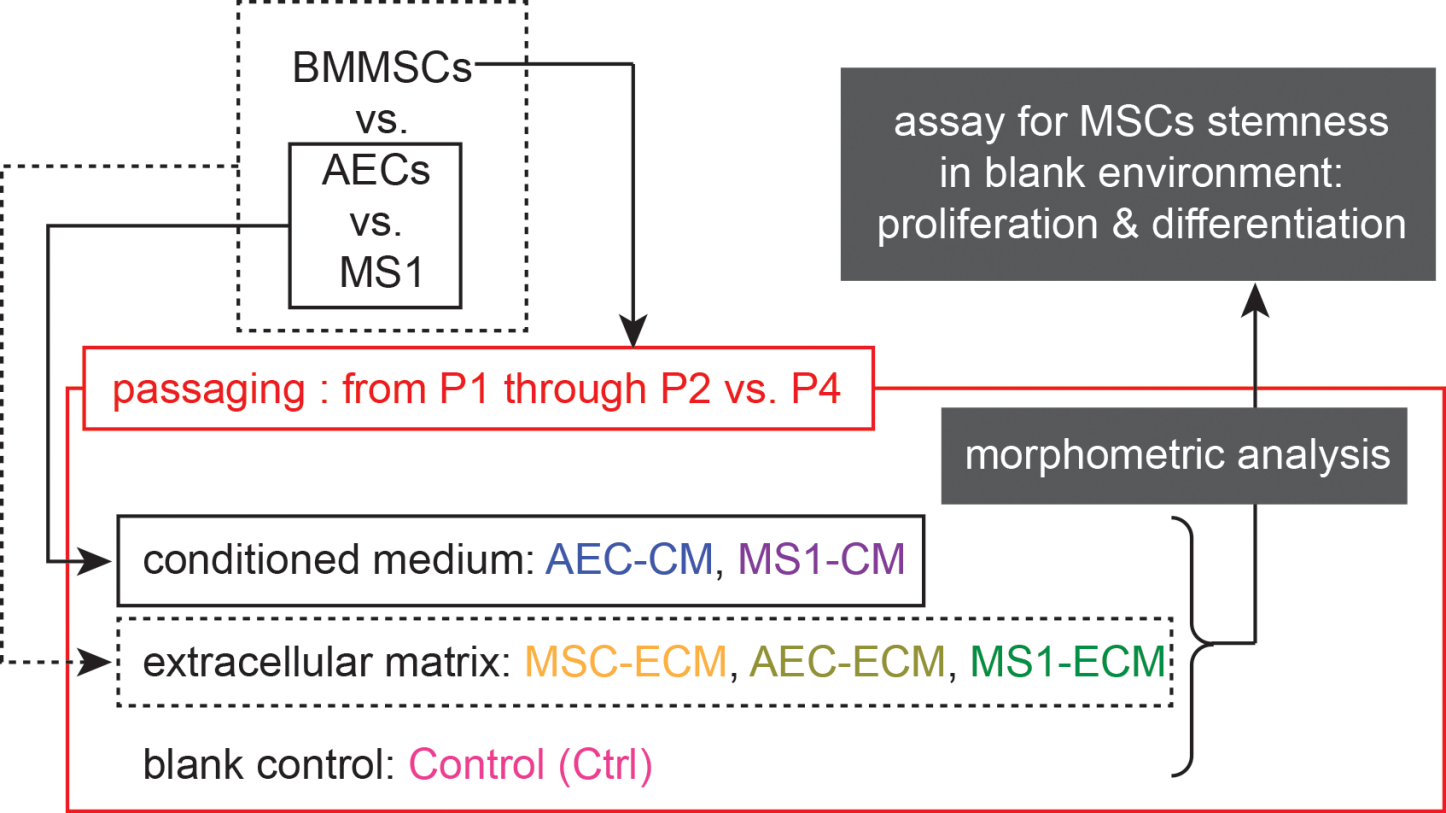
Diagramatic scheme of the experimental design. MSCs were passaged on 5 different niche-mimicking culture conditions and a blank standard culture condition as control group. For analysis, MSCs were detached from respective culture environment and settled in standard culture condition for proliferation and differentiation assessments.

To understand the different effects of ECs from large vessels and small vessels, two types of ECs were utilized in this study: primary mouse AECs as a large vessel-derived EC model, and MS1 mouse endothelial cell line representing the small vessel ECs. In each type of endothelial cells, both ECM and soluble factors, acquired in the form of CM, were examined for their effects on BM-MSCs. Accordingly, 6 niche-mimicking culture conditions were established (Fig 1): (1) Control: standard culture condition without any special treatment; (2) AEC-CM: MSCs seeded and passaged on standard culture condition supplemented with AECs-derived CM; (3) MS1-CM: MSCs seeded and passaged on standard culture condition supplemented with MS1-derived CM; (4) MSC-ECM: MSCs seeded and passaged on MSCs-derived ECM as another control for ECM groups; (5) AEC-ECM: MSCs seeded and passaged on AECs-derived ECM; (6) MS1-ECM: MSCs seeded and passaged on MS1-derived ECM. After isolation, MSCs were maintained on each niche-mimicking condition during passaging, and were analyzed for the stemness (i.e. proliferation and differentiation assay) in identical culture condition (as in Control group) to avoid direct interference from various niche components.

### MSCs maintained on endothelial ECM appeared morphologically juvenile

**Fig 2:**
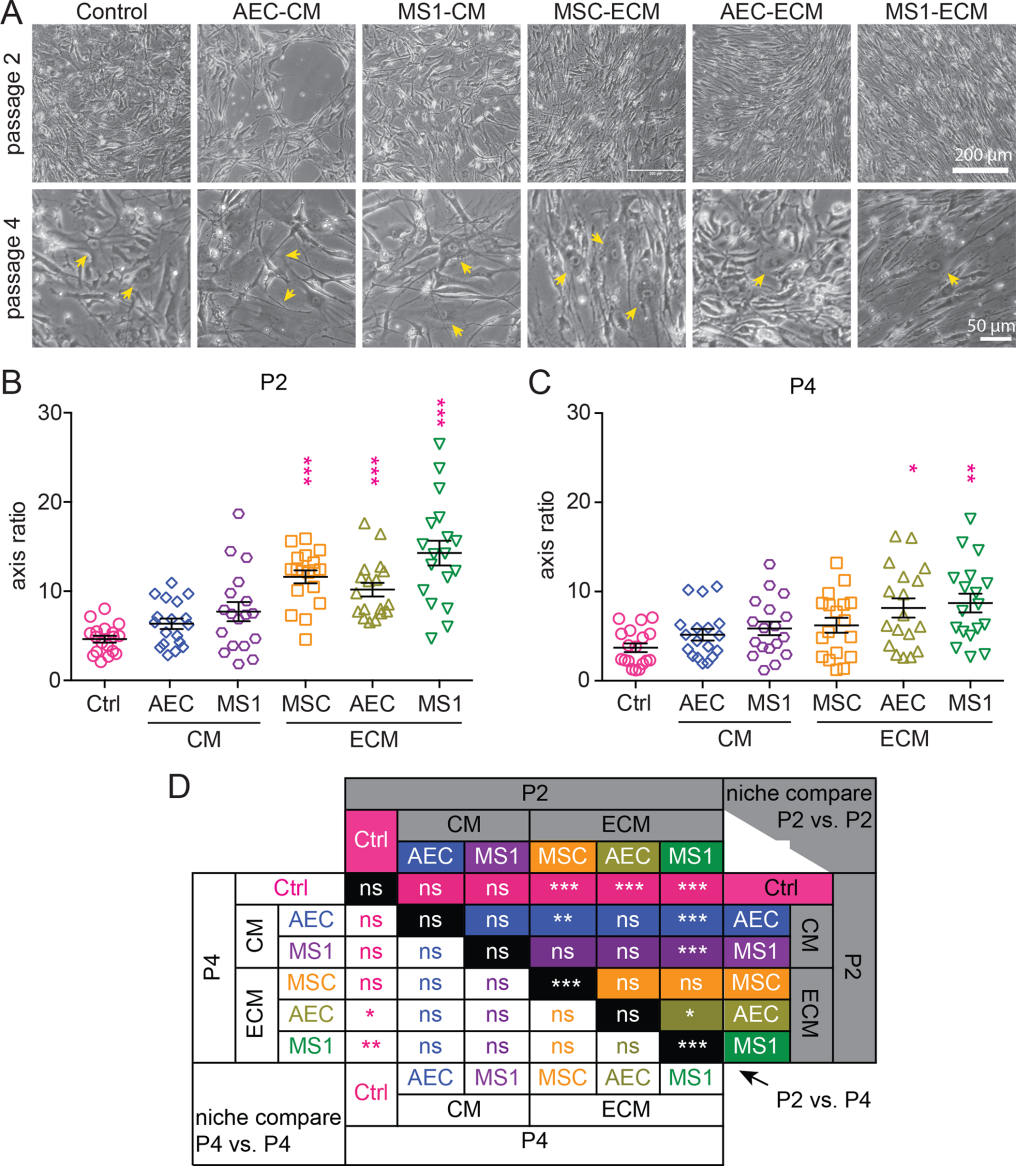
MSCs on endothelial ECM appeared more spindle-like morphology. (A) MSCs at passage 2 (upper panel, scale bar: 200 μm) and passage 4 (lower panel, scale bar: 50 μm) of each group showed various morphologies. MSCs on both AEC-ECM and MS1-ECM remained thin and slender. Flattened MSCs were observable at passage 4 (yellow arrows). (B-C) The lengths of 18 cells in each group were determined for the quantification of long/ short axis ratio in passage 2 (B) and 4 (C). MS1-ECM and AEC-ECM had significantly greater ratio compare to standard culture (pink asterisks) in both p2 and p4, while AEC-CM and MS1-CM remained comparative. (D) Detail results of statistics (One-way ANOVA with Tukey’s post-test; n = 18) were as listed. MSC-ECM showed drastic decrease of ratio from p2 to p4 (*p* < 0.001), leaving no significant difference compared to control. ns: not significant as *p* > 0.05; *: *p* < 0.05; **: *p* < 0.01; ***: *p* < 0.001.

Previous studies showed that MSCs lost their spindle shape when they lost their stemness [25, 27, 45]. In general, juvenile MSCs have obvious long and short axis, while senescent MSCs lose the polarity with flat and round shape. During passaging, MSCs among all groups continuously lost the spindle shape, but both endothelial ECMs tended to postpone this process. In passage 2, MSCs on AEC-ECM and MS1-ECM appeared spindle shape with apparent contrast of long and short axis aligning in a more uniformed orientation (Fig 2A). In passage 4, MSCs on both endothelial ECMs still maintained spindle shape with occasional flattened cells (arrows in Fig 2A), while many more cells started to increase in size and flatten in other groups, especially in control group (Fig 2A). In passage 2, all ECM groups had significantly (*p* < 0.001) greater ratio compared to control (Fig 2B and 2D). In addition, cells on MS1-ECM had greatest ratio and is significantly different compared to all other groups except for MSC-ECM group, while cells in AEC-CM and MS1-CM showed no difference compared to control group (Fig 2B and 2D). In passage 4, both AEC-ECM and MS1-ECM groups were significantly different (*p* < 0.05 and *p* < 0.01, respectively) compared to control group, while other groups showed no significant difference compared to control group (Fig 2C and 2D). Although MSC-ECM group was significantly different in their morphology compared to control group in passage 2 (Fig 2B and 2D), the long/short axis ratio rapidly declined and left no significance compared to control at passage 4 (Fig 2C and 2D). This finding indicated that MSCs were not fully self-sustainable for stemness maintenance. Both CM groups showed no effect on the morphology of MSCs. On the contrary, both endothelial ECM better maintained the spindle shape of MSCs, especially the MS1-ECM, in which the ratio of the MSCs remained greatest in both passage 2 and passage 4 (Fig 2).

### MS1-ECM mitigated the loss of proliferation potential of MSCs

**Fig 3:**
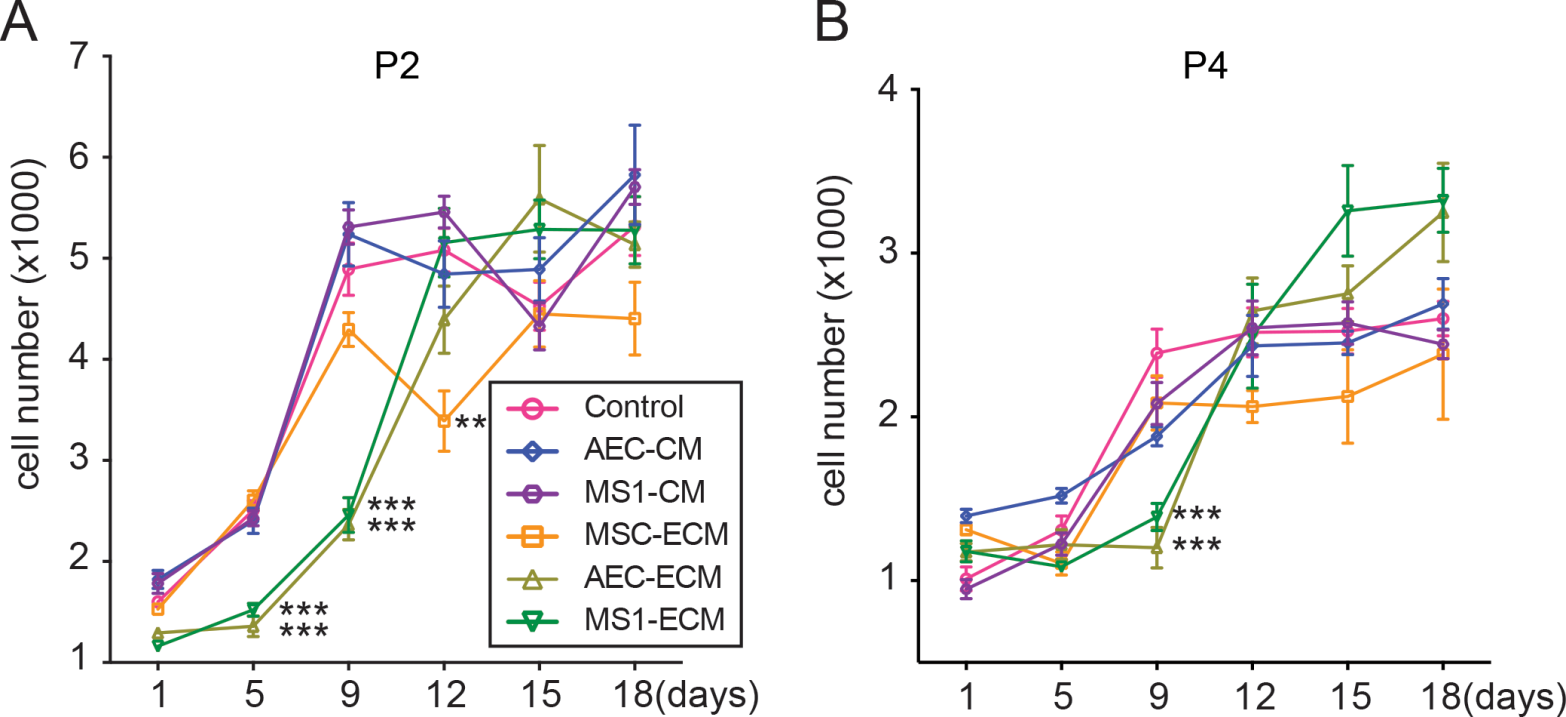
Endothelial ECM sustained MSCs proliferation capacity. MTT assay showed that in both passage 2 (A) and passage 4 (B), MSCs maintained on AEC-ECM and MS1-ECM expressed inactive cell cycle in the first 9 days (asterisks indicated statistical significance relative to the control group) after disengaged from the niche-mimicry environment. Meanwhile, the proliferative capacity was not compromised. Data from day 1 were not statistically analyzed. Detail results of statistics (One-way ANOVA with Tukey’s post-test; n = 8) were listed in S1–S5 Tables. **: *p* < 0.01; ***: *p* < 0.001.

To evaluate the proliferation potentials, MSCs passaged on respective culture conditions were seeded on the standard condition and assessed for proliferation by MTT assay. A total of 1,500 cells were seeded into 96-well plates with 8 replicates for each group. The numbers of cells were calculated at 1, 5, 9, 12, 15 and 18 days of incubation. In passage 2, during the first 9 days of incubation, MSCs on both AEC-ECM and MS1-ECM showed slower cell cycle and the cell numbers were significantly lower when compared to all other groups (*p* < 0.001 on both day 5 and 9) (Fig 3A) (S1 Table). All other groups reached plateau phase at 9th day of incubation, and both CM groups have significantly more cell numbers compared to all ECM groups but not the control group (Fig 3A) (S1 Table). However, during day 9 to 12, MSCs on AEC-ECM and MS1-ECM underwent excessive proliferation and reached plateau phase at day 12 or 15 resulting in comparable final cell counts similar to other groups (Fig 3A) (S2 Table). On day 18, only the cell count of AEC-CM was significantly greater than MSC-ECM (*p* < 0.05) (Fig 3A) (S3 Table). These results indicated that endothelial ECMs did not disrupt the proliferative potential of MSCs, although the initiation of active proliferation was delayed after disengaged from the endothelial ECM.

Similar phenomenon could be observed in passage 4. Both endothelial ECMs kept MSCs in slower cell cycle in the first 9 days, and entered progressive doubling afterwards (Fig 3B) (S4 Table). All other groups reached plateau phase at day 9 with a much lower levels of cell counts (ranged from AEC-CM 1,881 to Control 2,389 cells) compared to passage 2 (ranged from MSC-ECM 4,296 to MS1-CM 5,310 cells) (Fig 3A and 3B). Similarly, there was no significant difference after 12 days of culture among all groups with the exception of MSC count in MS1-ECM, which is significantly greater than MSC-ECM group on day 15 (*p* < 0.01) (Fig 3B) (S5 Table). Interestingly, in comparing the cell numbers between all passage 2 groups and all passage 4 groups at day 18, only AEC-ECM and MS1-ECM from passage 4 remained at comparable levels to MSC-ECM from passage 2 (Fig 3A and 3B) suggesting that the proliferative capacity of MSCs were better retained on endothelial ECMs.

In contrast to the slower initiation of proliferation in MTT assay, the endothelial ECM did not hinder the *ex vivo* expansion after MSCs were isolated. During passaging, MSCs were cultured on individual niche-mimicking conditions with the seeding density of 5×10^4^ cells per cm^2^, approximately 50% of confluence, and were passaged every 48 hrs when MSCs of all groups reached confluence. The comparison of cell counts demonstrated that more MSCs were harvested from both AEC-ECM and MS1-ECM than control group (*p* < 0.05) (Table 2).

**Table 2:** Harvested MSC count on each culture condition.

Group	Harvested cells (10^6^ cells)	Different from control?
Control	1.49 ± 0.04	
MSC-ECM	2.02 ± 0.24	No
AEC-ECM	2.30 ± 0.15	Yes (*p* < 0.05)
MS1-ECM	2.24 ± 0.46	Yes (*p* < 0.05)

All numbers of harvested MSCs (passage 2) were normalized to the initial seeding number (1×10^6^). All data were presented as mean ± SEM.

### MS1-ECM preserved tri-lineage differentiation potential of MSCs

**Fig 4:**
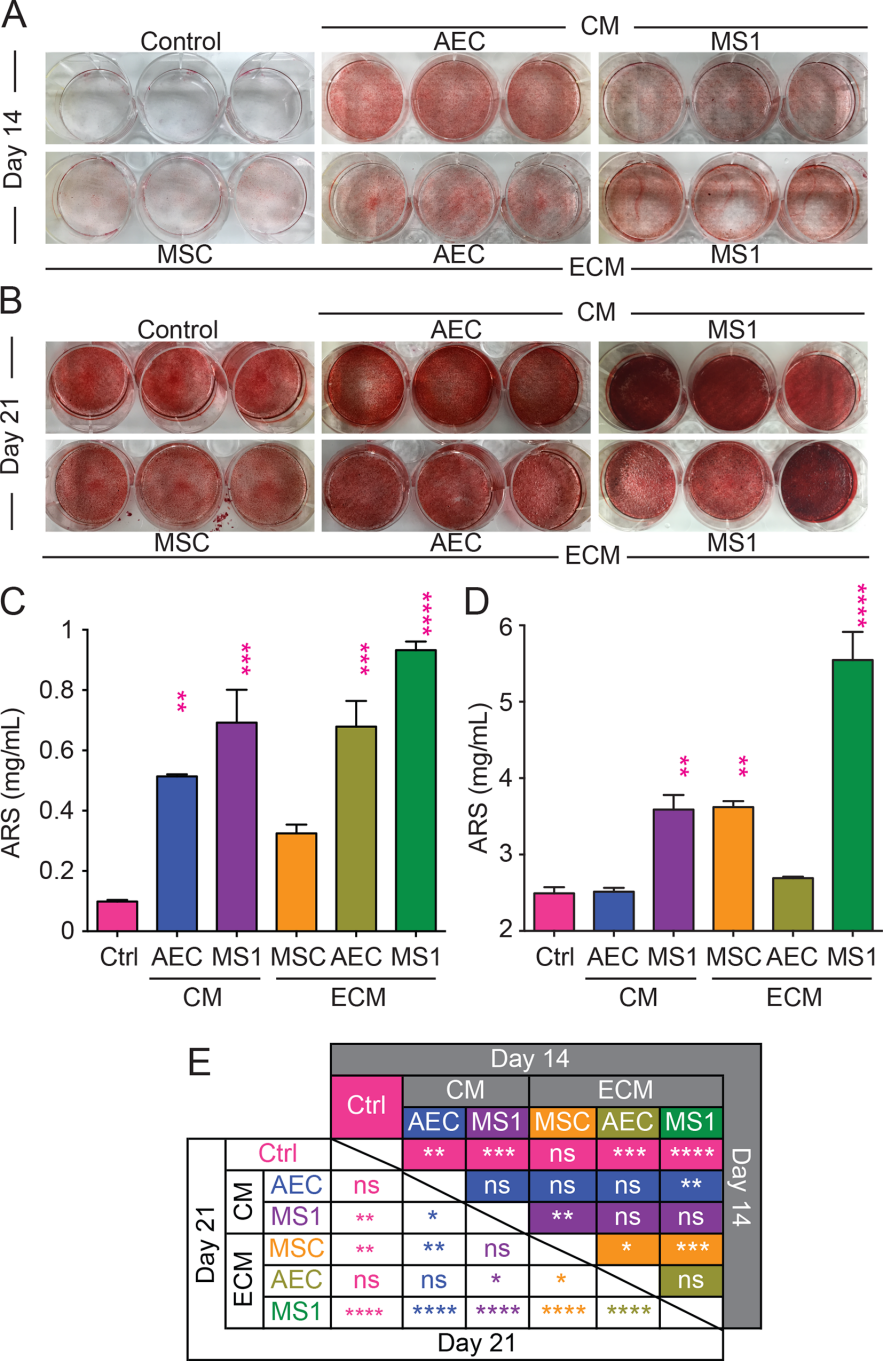
MSCs on MS1-ECM performed privileged osteogenic differentiation. MSCs from passage 3 were introduced to ostegenic induction medium for 14 (A) and 21 (B) days and underwent ARS staining to evaluate the calcium precipitation. In the quantification data of calcium precipitation after 14 (C) days and 21 (D) days of induction, although multiple groups show statistical significance compared to the control group (pink asterisks), MS1-ECM group emerged to show significantly more calcium precipitation compared to all other groups after 21 days of induction as listed in the detail statistical results (E). ns: not significant as *p* > 0.05; *: *p* < 0.05; **: *p* < 0.01; ***: *p* < 0.001.

Under proper stimulation, MSCs should be able to differentiate into osteogenic, adipogenic and chondrogenic progenies. Osteogenic induction promotes MSCs to deposit inorganic minerals and calcium precipitation can be regarded as a functional state of osteogenic differentiation. MSCs expanded on respective niche-mimicking conditions for two passages, and were introduced to osteogenic stimulation after disengaged from the niche-mimicking conditions. After 14 and 21 days of induction, calcium precipitations were visualized by ARS staining (Fig 4A and 4B). Quantification of ARS staining after 14 days of stimulation showed that osteogenic potential was significantly augmented in MSCs expanded on endothelial ECM and CM, while MSCs expanded on MSC-ECM showed a comparative calcium precipitation to control (Fig 4A, 4C and 4E). Comparisons among endothelial-derivative groups showed that MS1-ECM preserved the most osteogenic potential, albeit significant differences lied only against AEC-CM (*p* < 0.05) (Fig 4C and 4E). Interestingly, the trend that MS1-ECM preserve the most osteogenic potential of MSCs became significantly observable after 21 days of induction that MSCs expanded on MS1-ECM showed significantly higher calcium precipitation compared to all other groups (*p* < 0.001) (Fig 4D and 4E). On the other hand, the osteogenic potential of AEC-derivatives groups seemed to reach an early saturation and showed no significant difference compared to the control group (Fig 4D and 4E). These data indicated that MSCs expanded on MS1-ECM could retain the most osteogenic potential.

**Fig 5:**
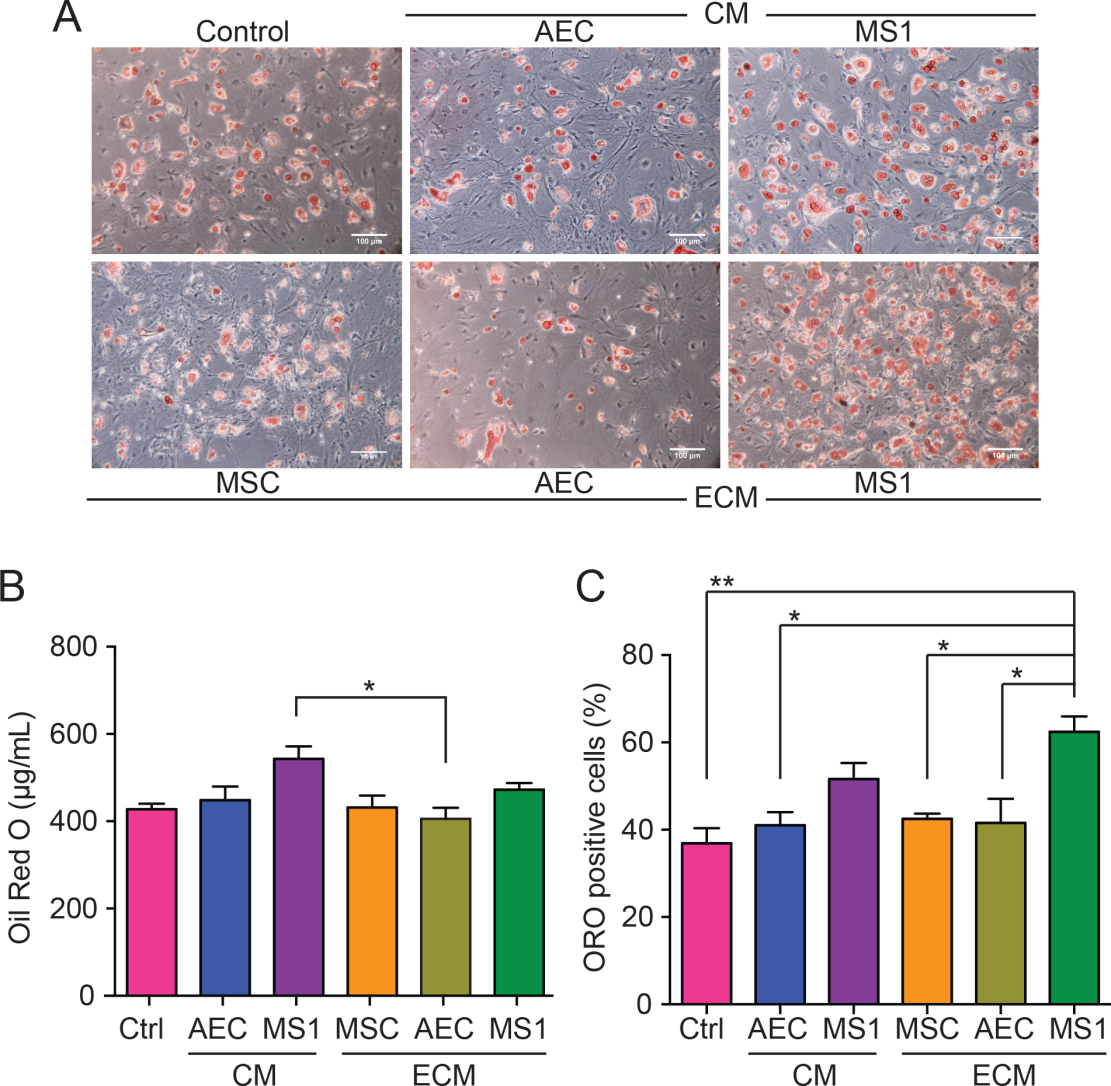
MS1-ECM reserved adipogenic plasticity of MSCs. (A) Lipid droplets could be observed after 7 days of induction. Scale bar: 100 μm (B) Oil Red O staining demonstrated each group had similar amount of neutral lipid accumulation. Only MS1-CM had greater lipid accumulation than AEC-ECM. (C) MS1-ECM had higher population of cells with adipogenic potential. *: *p* < 0.05; **: *p* < 0.01.

Another monumental characterization of MSCs is their ability to differentiate into adipogenic lineage and accumulate lipid droplets inside cells with the administration of adipogenic induction medium. Similar to osteogenic induction, MSCs that were expanded on respective niche mimicry for two passages were seeded in standard culture condition with adipogenic stimulation. After 7 days of induction, Oil Red O staining exhibited neutral lipids accumulation in the cells (Fig 5A). Total neutral lipids quantification showed no significant difference among all groups with the exception of MS1-CM group that had greater lipid accumulation compared to AEC-ECM group (*p* < 0.05) (Fig 5B). It is noticeable that the lipid accumulation in each cells varied dramatically (Fig 5A) suggesting that the commitment of adipogenic lineage is sporadic for MSCs. Acccordingly, the populations of MSCs that were able to commit to adipogenic lineage were assessed by counting the number of Oil Red O positive cells. Despite the fact that total lipid accumulation was comparable, MS1-ECM group had significantly higher Oil Red O positive cells compared to control (*p* < 0.01), MSC-ECM, AEC-ECM and AEC-CM (*p* < 0.05) (Fig 5C). This result strongly suggested that MS1-ECM could constitute a niche-like environment and maintain MSCs in a more primitive and uncommitted state.

**Fig 6:**
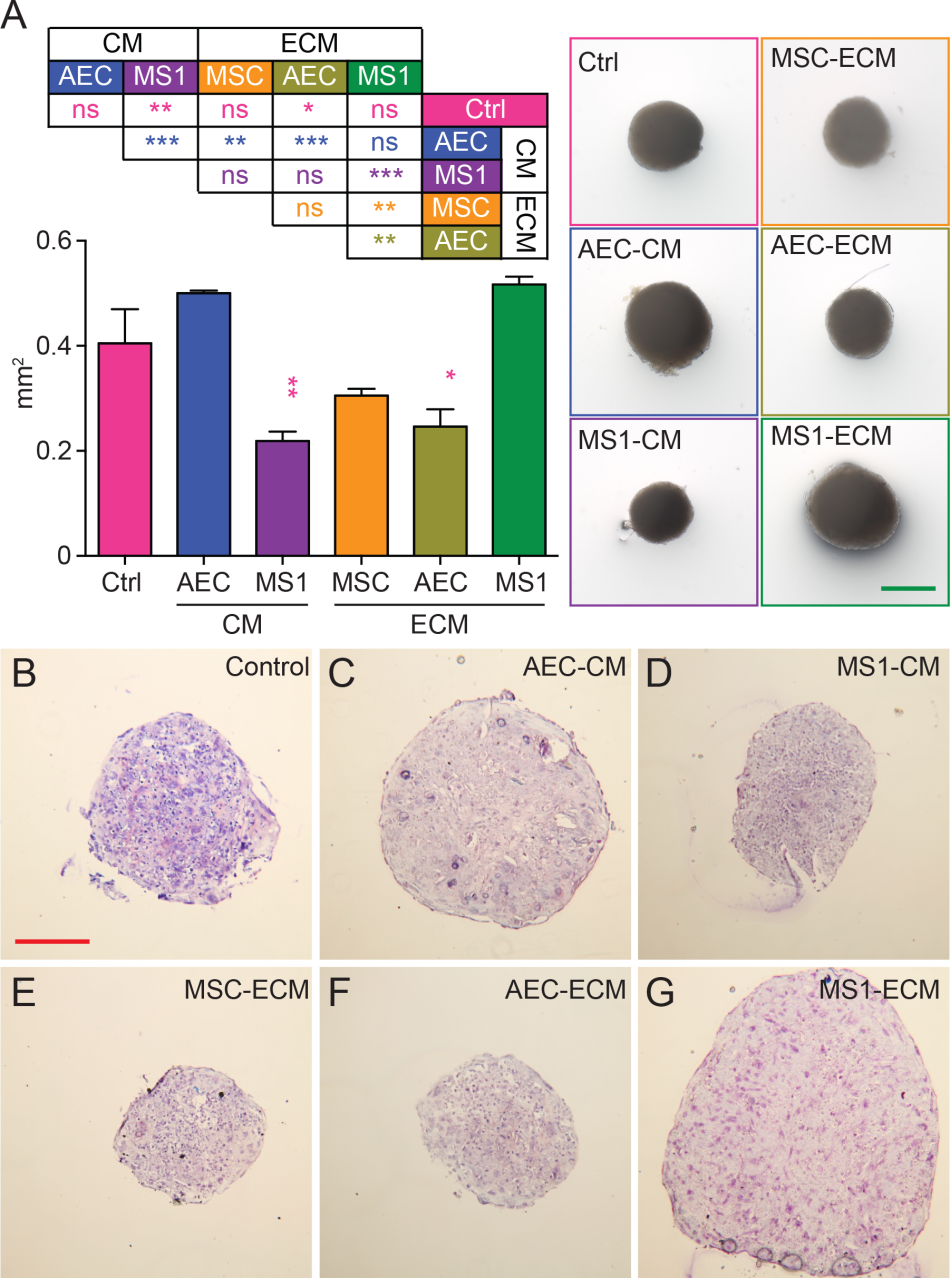
Endothelial derivative altered chondrogenic potential. (A) The projected area of sphere was calculated. Endothelial derivatives showed variable effects on chondrogenic potential. (ns: not significant as *p* > 0.05; *: *p* < 0.05; **: *p* < 0.01; ***: *p* < 0.001) Green scale bar: 500 μm (B) Bright field photos of Toluidine Blue O staining of cell aggregates showed purple hue for GAGs content. MS1-ECM had more purple clusters while control group had little purple stains. Red scale bar: 200 μm.

To evaluate the potential for chondrogenic differentiation, MSCs were centrifuged to generate self-aggregated spheres and incubated in chondrogenic medium. After 21 days, the sizes of aggregates were measured and aggregates derived from MS1-ECM and AEC-CM groups showed significantly bigger sizes compared to other derivative-treated groups (Fig 6A). On the other hand, the sizes of AEC-ECM (*p* < 0.05) and MS1-CM (*p* < 0.01) groups were significantly smaller than control (Fig 6A). Since the accumulation of glycosaminoglycans (GAGs) are a general indicator to evaluate chondrogenic differentiation [46], paraffin sections of aggregates were stained with Toluidine Blue O to evaluate GAGs component within the sphere (Fig 6B–6G). In the control group (Fig 6B), several parts of the sphere showed cracked gaps with loosely connection between cells indicating that the condensation of cell aggregate was not well proceeded. In addition, the section showed large distribution of blue stain (nucleic acids) with some purple (GAGs) dispersed around the center indicating that only little GAGs had accumulated during the induction. In contrast to all other groups, the sphere of MS1-ECM group (Fig 6G) was compact with smooth border and most abundant purple stains scattering throughout the sphere indicating that the MSCs in MS1-ECM group possessed the best chondrogenic potential.

**Fig 7:**
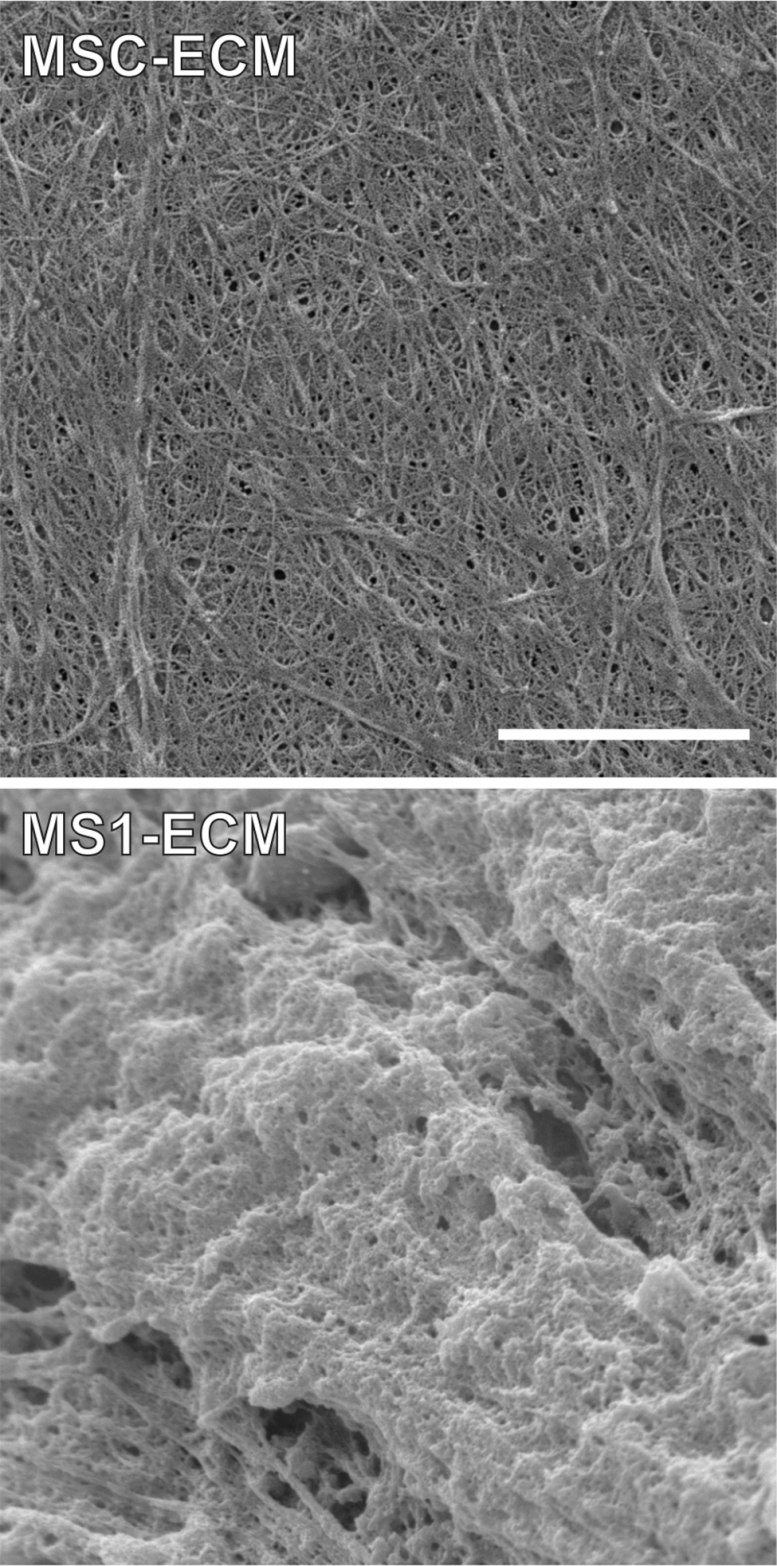
MS1-ECM showed distinct organization from MSC-ECM. ECMs derived from MS1 cells and MSCs were observed under a SEM. Compared to MSC-ECM, MS1-ECM showed a thicker and more condensed framework with more uniformly oriented organization, while MSC-ECM was thinner and loosely organized without any apparent orientation. Scale bar: 4 μm.

When observed under the scanning electron microscope, MS1-ECM showed a thicker appearance with condensed and well-aligned fibrillar fabrics, while MSC-ECM showed a thinner appearance lacking any apparent orientation (Fig 7).

### MSCs expanded on MS1-ECM showed a better immune-modulatory potential

**Fig 8:**
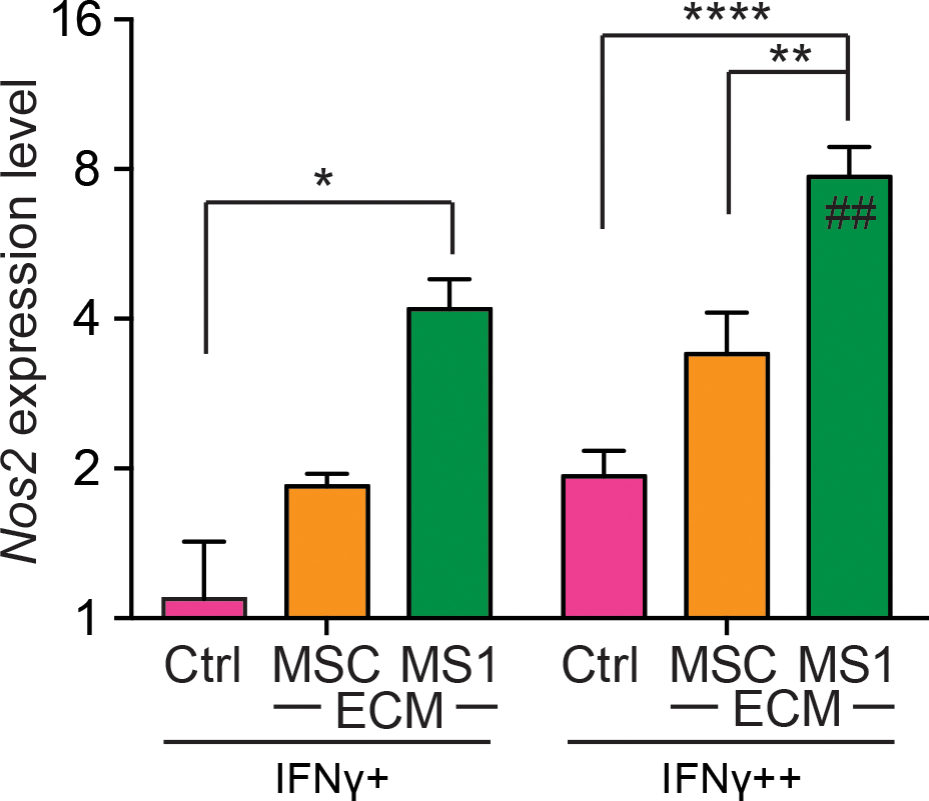
MSCs expanded on MS1-ECM express highest level of *Nos2* in response to interferon gamma. The expression level of iNOS (*Nos2*) was evaluated by real-time qPCR. Passage 3 of MSCs expanded on MS1-ECM showed the strongest *Nos2* expression in response to the treatment of a low dose interferon gamma (IFNγ+), while this trend became more significant at a high dose interferon gamma (IFNγ++) treatment. Note that only MS1-ECM group showed a significant increased *Nos2* expression in response to the increase of interferon gamma dose (##). Data was calibrated with *Gapdh* then standardized with control at low dose of interferon gamma. *: *p* < 0.05; **: *p* < 0.01; ****: *p* < 0.0001; ##: *p* < 0.01 relative to low dose.

One of the key beneficial effects of MSCs came from the inflammation-responsive immune-modulatory activity [47–49], while MSCs could lose this activity quickly through passaging [30]. In mouse, this inflammation-induced immunosuppressive activity of MSCs was mediated by inducible nitric oxide synthase (iNOS or *Nos2*) [50]. We therefore evaluated the expression of iNOS (*Nos2*) in MSCs after disengaged from MSC-ECM or MS1-ECM and subjected to interferon gamma (IFNγ). Interestingly, the iNOS expression levels in MSCs were comparable among control, MSC-ECM and MS1-ECM groups and between high and low doses of interferon-γ at passage 2 (data not shown), but at passage 3, MSCs expanded on MS1-ECM showed the most responsive surge of iNOS expression with a dose-dependent manner (Fig 8) suggesting that MS1-ECM effectively preserved the immune-modulatory potential of MSCs.

### MS1-ECM triggered a higher H3K27me3 content and lower KDM6B expression

**Fig 9:**
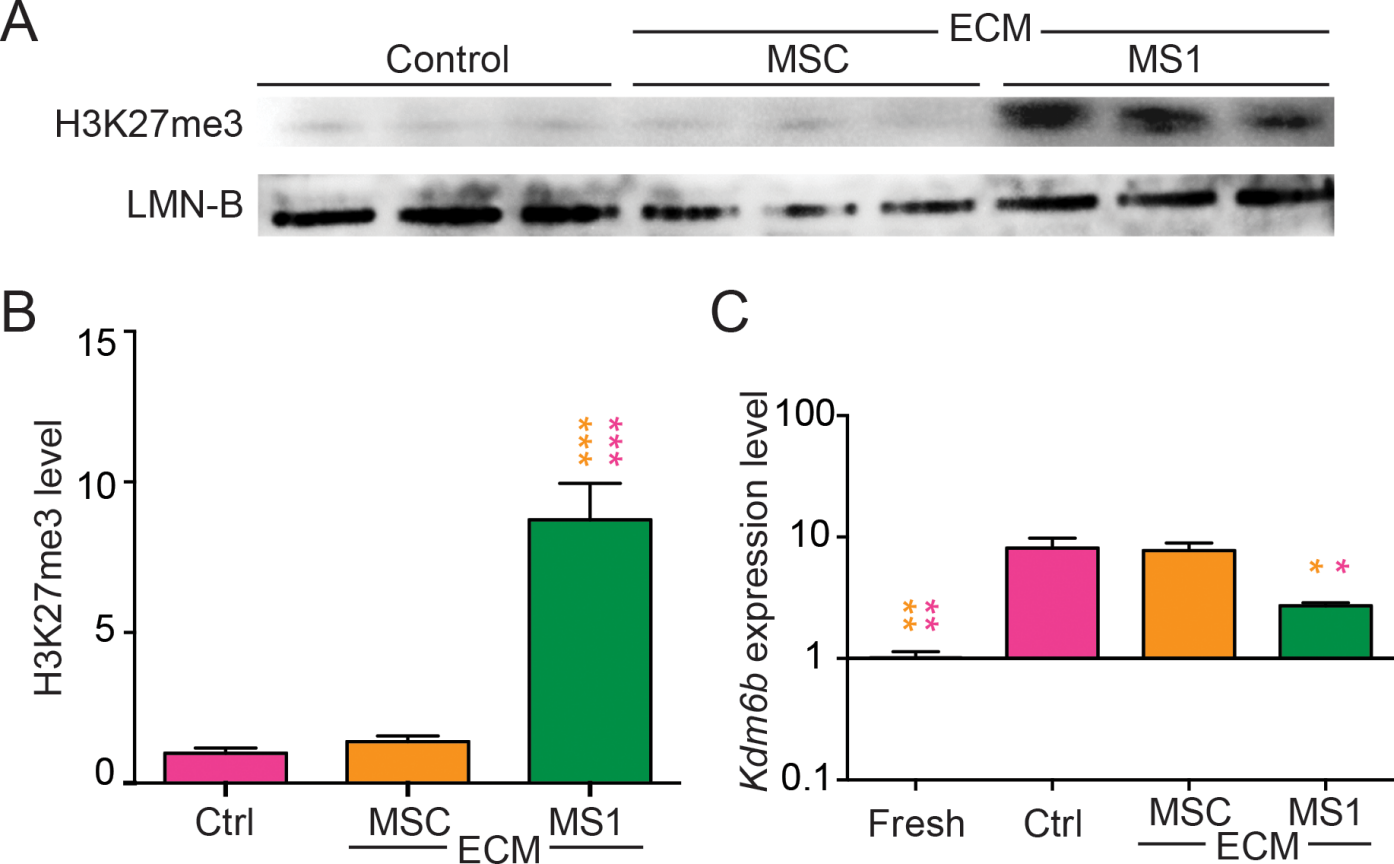
MSCs on MS1-ECM had higher trimethyl lysine 27 on histone 3. (A) H3K27me3 levels of control, MSC-ECM and MS1-ECM were investigated with lamin B (LMN-B) as loading control. (B) Quantification of global H3K27me3 levels calibrated with lamin B showed that MSCs on MS1-ECM had significantly higher H3K27me3 compared to control (pink asterisks) and MSC-ECM (orange asterisks). (C) Quantitative RNA expression level of *Kdm6b* showed that, similar to freshly isolated MSCs (Fresh), MSCs on MS1-ECM had significantly lower *Kdm6b* expression than control (pink asterisk) and MSC-ECM (orange asterisk) groups. Data were calibrated with *Gapdh* then standardized with expression level of purified group. * *p* < 0.05; ** *p* < 0.01; *** *p* < 0.001.

It has been revealed that histone H3 modification has huge impact on stem cell-activities [51]. Accordingly, MSCs maintained on MS1-ECM had significantly higher global H3K27me3 compared to control and MSCs on MSC-ECM (*p* < 0.001) (Fig 9A and 9B). In general, higher global H3K27me3 represented general suppression in gene transcription [52], which could explain the primitive fate commitment and greater differentiation plasticity of MSCs expanded on MS1-ECM.

The increase in inhibitory chromatin signature H3K27me3 could be potentially due to the up-regulation of H3K27 methyltransferase *Ezh2*, or the down-regulation of H3K27 demethylases *Kdm6a* and *Kdm6b*. The gene expression levels of these enzymes were evaluated in freshly isolated MSCs (passage 0) alongside with control (passage 3), MSC-ECM (passage 3) and MS1-ECM (passage 3). Both the expression levels of *Ezh2* and *Kdm6a* among all groups were with no statistical significance (data not shown). Interestingly, the expression levels of *Kdm6b* in MSCs of both control (*p* < 0.01) and MSC-ECM (*p* < 0.01) groups were significantly increased when compared to the freshly isolated MSCs (Fig 9C). Moreover, *Kdm6b* expression in MSCs on MS1-ECM not only was comparable to the freshly isolated MSCs, but also was significantly lower than both control (*p* < 0.05) and MSC-ECM (*p* < 0.05) groups (Fig 9C). This result suggested that MSCs possibly underwent vigorous demethylation at H3K27 once isolated from the endogenous environment, while MS1-ECM could retain the stemness of MSCs by triggering a higher H3K27me3 accumulation and a lower *Kdm6b* expression, which was slightly up-regulated in MS1-ECM with no significant difference compared to the freshly purified MSCs.

## Discussion

The scarce nature of MSCs is the fundamental reason why *ex vivo* expansion is obligatory before they can be applied for cell therapies. Previous studies have successfully improved MSCs proliferation and osteogenic differentiation with MSCs-derived ECM. MSCs cultured on ECM derived from early passage marrow stromal cells had better colony-forming efficiency, showed greater differentiation potential and retained more molecular phenotypes compared to those on standard plastic dish or dish coated with either fibronectin or collagen I [40, 53]. MSCs cultured on marrow stromal cell-derived ECM with porous titanium fiber scaffold significantly enhanced their proliferation and osteogenic potential compared to those cultured either only on scaffold or only on ECM [54]. When aged MSCs maintained on ECM derived from marrow stromal cells with younger age, the deteriorating proliferation and osteogenic capacity in concert with telomerase activity could be rescued [55]. Co-transplantation of MSCs and MSCs-derived ECM facilitated wound healing and osteo-regeneration in a mouse calvarial bone fracture model [56]. Although our study showed that MSC-ECM did bring about a better osteogenic potential compared to standard culture procedure (Fig 4), the proliferation potential was not significantly different (Fig 3). This discrepancy was possibly due to the fact that the preparation ECM of other studies was derived from the mixture of freshly isolated bone marrow cells (passage 1 to 2), and the MSCs for ECM preparation in this study were not only from enriched MSCs but also at passage 2 or 3. Previous studies show that MSCs gradually lose their stemness through serial passages [53] and that cell therapy using higher passaged MSCs compromised the results [30]. These lines of evidence and ours not only indicated that ECM profoundly affect the stemness of MSCs and suggested that MSCs are not capable of orchestrating their very own stem cell niche.

Previous studies showed that, the sizes and numbers of MSC colonies were increased when cultured with HUVECs-derived CM [57], however the MSCs were less proliferative when co-cultured in contact with HUVECs [36]. It is interesting that different derivatives (CM vs. ECM) from the same cells exerted opposite influences on MSCs. Since endothelial soluble factors promoted MSCs proliferation, the quiescence might be caused by direct contact between MSCs and HUVECs or between MSCs and HUVEC-derived ECM or both. Accordingly, our study reported that both AEC-CM and MS1-CM promoted MSCs proliferation in the first 9 days of culture in MTT assay, while MSCs remained slow cell cycle if previously cultured on endothelial ECM (Fig 3). However, harvested cell count during passaging demonstrated that more MSCs were harvested on endothelial ECM than conventional culture condition (Table 2). If ECM provided relatively quiescent signals, why would this niche mimicry end up contributing more cells? The passaging seeding density during passaging in our study was 5×10^4^ cells per cm^2^, approximately 50% of confluence. Passaging took place after 48 hours of culture and generally MSCs from all groups reached confluence by this time. Previous study reported that population doubling of BM-MSCs ranges between 20 to 40 hours [37], and thus the MSCs possibly went on more than one replication in our study. Since MSCs on endothelial ECM appeared slenderer with organized orientation, the petri dish might be capable of accommodating more cells than other groups. This might explain why more MSCs were harvested on endothelial ECM, and it could be a more efficient way to amplify MSCs without undergoing excessive passaging. No matter what mechanism was behind this phenomenon, the proliferative potential and efficiency was not compromised in endothelial ECM.

Previous studies demonstrated that BM-MSCs cultured with ECs improved osteogenesis [58, 59]. However, when BM-MSCs cultured with HUVECs or HUVECs-derived CM, the adipogenic potential reduced or maintained comparable [36, 57]. One possible reason is that the origins of MSCs predispose the differentiation preferences, and osteogenesis is likely the default fate of BM-MSCs [60, 61]. It is reported that periosteum-derived MSCs had greater osteogenic and chondrogenic potential with weaker adipogenic potential, while adipose-derived MSCs had superior adipogenic differentiation with feeble osteogenic and chondrogenic differentiation [62]. In our study, aside from the fact that all endothelial derivatives enhanced osteogenesis, MS1-CM elevated total neutral lipid accumulation compared to AEC-ECM, while MS1-ECM had highest Oil Red O-stained population (Fig 5B and 5C). MSCs maintained on MS1-ECM also showed better chondrogenic differentiation (Fig 6). These findings indicated that small vessels-derived ECs possess a distinctive potential to counteract the endogenous lineage limitation and preserved the differentiation plasticity of BM-MSCs compared to AECs and HUVECs. Accordingly, previous study demonstrated ubiquitous existence of pericytes surrounding capillaries (< 10 μm) and arterioles (10–100 μm), where these pericytes expressed MSCs characterization [33], and BM-MSCs seemed to dwell in perivascular location of small vessels [32, 63].

Furthermore, since MSCs expanded on MS1-ECM duplicated more times than standard culture condition (Table 1), the preservation of differentiation plasticity was probably not because MSCs underwent fewer replications on MS1-ECM, but instead, it was because of signals given from MS1-ECM. Our results indicated that ECM derived from small vessels ECs but not large vessels ECs can better mimic the niche microenvironment of MSCs and preserve MSCs stemness, which was in accordance with previous studies that BM-MSCs anchored on vascular wall of sinusoidal ECs [32, 63]. ECs from large and small vessels had diverse gene expression patterns. Large vessels ECs tended to express genes involving in biosynthesis of ECM including fibronectin, collagen 5α1, collagen 5α2 and osteonectin, which were likely related to thick wall structures around the endothelial cells of large vessels, while small vessels ECs inclined to express genes concerning basement membrane proteins, such as laminin, collagen 4α1, collagen 4α2 and ECM-interacting proteins like a collection of integrins [64]. Those differences in gene expression profiles might contribute to variable ECM composition between large vessels and small vessels ECs, and in turn affect the MSCs stemness.

The trimethylation of H3K27 usually correlates with silent heterochromatin state [52] and is indispensable for the stemness maintanence of embryonic stem cells [65–67]. This chromatin signature is catalyzed by polycomb repressor complexes 2 (PRC2), a complex consists of the histone methyl-transferase EZH2 (enhancer of zeste homologue 2) [68], while demethylation is performed by KDM6A (lysine (K)-specific demethylase 6A, also known as UTX) and KDM6B (lysine (K)-specific demethylase 6B, also known as JMJD3). The two demethylases work specifically and independently to “erase” methyl mark on lysine 27 residue of histone H3 [69]. The regulation of H3K27 mark has been proved crucial for the lineage commitment of MSCs. For example, BMP-driven osteogenic differentiation upregulated KDM6B expression, while knockdown of KDM6B disrupted *in vitro* mineralization and lowered osteogenic genes. Conversely, overexpression of KDM6B augmented mineralization by removing the H3K27me3 chromatin signature at the promoter region of osteogenic related genes [70]. In addition, overexpression of EZH2 contributed to higher adipogenic potential in concert with higher adipogenic genes like PPAR-γ, C/EBP-α and adipsin, while giving rise to lower osteogenic potential and transcription levels of osteogenic genes like RUNX2, ostepontin and osteocalcin. In contrast, overexpression of KDM6A, the enzyme antagonizes EZH2 activity, resulted in adipogenesis suppression and enhancement of osteogenesis [71]. In our study, we found that higher H3K27me3 chromatin signature was correlated with higher stemness of MSCs as the MSCs expanded on MS1-ECM with most prominent H3K27me3 signature. In line with this finding, we also found a significantly lower *Kdm6b* level in MS1-ECM group compared to control and MSC-ECM groups, while this low *Kdm6b* level is comparable to that in freshly isolated BM-MSCs (Fig 9C) suggesting that MS1-ECM retained the high H3K27me3 chromatin signature by maintaining a low *Kdm6b* level and thereby preserved the stemness of MSCs. It would also be interesting to profile other global histone modifications such as H3K4me3, H3K9me3 and histone acetylation among control, MSC-ECM and MS1-ECM as well as freshly isolated MSCs to obtain a better picture of the influence of MS1-ECM on MSC chromatin signature.

ECM from small vessels-derived ECs had better outcome on MSCs stemness preservation than MSCs-derived ECM and AECs-derived ECM. In our SEM images, MS1-ECM showed thicker and more condensed fabrics with uniformly oriented organization compared to MSC-ECM (Fig 7), and this result was well correlated to the better uniformly orientation and slenderer shape of MSCs expanded on MS1-ECM. Many factors of ECM could influence stem cells, such as elasticity, mechanical signals transduction, glycosaminoglycans (GAGs) and morphogens trapped in ECM [72, 73]. Tension of ECM and nanotopography alter the focal adhesion ensuing changes in cell shape and in turn affect MSCs osteogenic differentiation [74]. ECM stiffness can also act as a regulator on MSCs fate. The stiffness of growing surface determines the quiescence, plasticity and lineage commitment of MSCs [75, 76]. ECM components include a great deal of collagen, fibronectin, laminin and GAGs, which could potentially trap morphogens and affect stem cell commitment [40]. It is likely that ECM-derived from different cells have distinct compositions, and might also direct MSCs activities. This speculation requires further investigation, but the insoluble components of mesh network and soluble bioactive factors that are sequestered in the small vessels-derived ECM might be the key effectors to preserve MSCs stemness.

Taken together, we reported small vessels-derived ECM (MS1-ECM) preserved juvenile morphology, proliferation capacity, and differentiation plasticity of MSCs, indicating that MS1-ECM preserves MSCs stemness. The possible mechanism is that MS1-ECM shaped MSCs a transcriptional inactivation chromatin signature with a higher H3K27me3 mark and a lower *Kdm6b* expression. In addition, the MS1-ECM also retained the inflammatory-induced immune-modulatory activity of MSCs even at a later passage. Our findings not only provide supportive evidence that MSCs reside in a perivascular niche, but also a novel approach for *ex vivo* expansion of MSCs.

## Supporting information

S1 Table Detail results of statistical analyses (One-way ANOVA with Tukey’s post-test; n = 8) on proliferation at Day 5 and 9 of passage 2. (DOC)

S2 Table Detail results of statistical analyses (One-way ANOVA with Tukey’s post-test; n = 8) on proliferation at Day 12 and 15 of passage 2. (DOC)

S3 Table Detail results of statistical analyses (One-way ANOVA with Tukey’s post-test; n = 8) on proliferation at Day 18 of passage 2 and 4. (DOC)

S4 Table Detail results of statistical analyses (One-way ANOVA with Tukey’s post-test; n = 8) on proliferation at Day 5 and 9 of passage 4. (DOC)

S5 Table Detail results of statistical analyses (One-way ANOVA with Tukey’s post-test; n = 8) on proliferation at Day 12 and 15 of passage 4. (DOC)
