# Supplementary material for: Endothelial-derived extracellular matrix ameliorate the stemness deprivation during ex vivo expansion of mouse bone marrow-derived mesenchymal stem cells
Source: PLoS One. 2017 Aug 30;12(8):e0184111. doi: 10.1371/journal.pone.0184111 (PMC5576725; doi:10.1371/journal.pone.0184111)
Supplement: S5 Table — (DOC) [file pone.0184111.s005.doc]

**S5 Table.** Detail results of statistical analyses (One-way ANOVA with Tukey’s post-test; n = 8) on proliferation at Day 12 and 15 of passage 4

| P4 | | | Day12 | | | | | | Day12 |
| --- | --- | --- | --- | --- | --- | --- | --- | --- | --- |
|  |  |  | Ctrl | CM | | ECM | | |  |
|  |  |  |  | AEC | MS1 | MSC | AEC | MS1 |  |
| Day15 | Ctrl | |  | ns | ns | ns | ns | ns |  |
|  | CM | AEC | ns |  | ns | ns | ns | ns |  |
|  |  | MS1 | ns | ns |  | ns | ns | ns |  |
|  | ECM | MSC | ns | ns | ns |  | ns | ns |  |
|  |  | AEC | ns | ns | ns | ns |  | ns |  |
|  |  | MS1 | ns | ns | ns | ** | ns |  |  |
|  | Day15 | | | | | | | |  |
